# Hypoxia-associated genes as predictors of outcomes in gastric cancer: a genomic approach

**DOI:** 10.3389/fimmu.2025.1553477

**Published:** 2025-03-10

**Authors:** Shuo Yang, Yuhao Jiang, Zhonghua Yang

**Affiliations:** ^1^ Department of Pediatric Surgery, Shengjing Hospital of China Medical University, Shenyang, China; ^2^ NHC Key Laboratory of Congenital Malformation, Shengjing Hospital of China Medical University, Shenyang, China

**Keywords:** stomach adenocarcinoma, hypoxia, single-cell RNA sequencing, prognostic model, transcription factor, qRT-PCR

## Abstract

**Objective:**

To investigate the effects of hypoxia-related genes in stomach adenocarcinoma (STAD) and construct an excellent prognostic model.

**Methods:**

RNA expression data and clinical details were retrieved from the TCGA and GEO database dataset. scRNA-seq analysis was conducted on primary gastric cancer samples from GSE183904. Cellular hypoxia status was predicted using the CHPF software. WGCNA and GO-BP/KEGG enrichment of module genes analyses were performed to identify gene modules associated with hypoxia and biological pathway enrichment. A prognostic model was developed employing the LassoCox algorithm. GES-1, AGS, BGC823, and MGC803 cell lines were obtained for qRT-PCR analysis to identify the expression of model genes.

**Results:**

Single-cell atlas within STAD delineated that most of neoplastic cells, fibroblasts, endothelial cells, and myeloid cells were hypoxic. Further analysis of neoplastic cell subpopulations identified four hypoxic subpopulations (H1-H4) and four non-hypoxic subpopulations (N1-N4), with H1 subpopulation had the highest degree of hypoxia. The prognostic model constructed by five H1-specific transcription factors EHF, EIF1AD, GLA, KEAPI, and MAGED2, was demonstrated efficacy in predicting overall survival (OS), with significantly worse OS in high-risk patients. qRT-PCR analysis determined the higher expression level of five H1-specific transcription factors in gastric cancer cell lines than that in normal gastric epithelial cell line.

**Conclusion:**

Hypoxia exerts a profound influence on STAD due to the overexpression of hypoxic cellular subpopulations-specific transcription factors EHF, EIF1AD, GLA, KEAPI, and MAGED2. The novel prognostic model developed by these hypoxia-associated genes presents a novel approach to risk stratification, exhibiting an excellent prognostic value for STAD patients.

## 1 Introduction

Stomach Adenocarcinoma (STAD) ranks as one of the most prevalent cancers globally, characterized by significantly high incidence and fatality rates (1). Gastric cancer exhibits a notably high incidence in China on a global scale (2). The prognosis for STAD remains poor, featuring a five-year survival rate of 6% in the metastatic setting that is intimately tied to the tumor’s aggressive nature, heterogeneity, and resilience to therapeutic interventions (3).

In recent periods, the influence of hypoxia in tumor development has attracted widespread focus. There is substantial proof that the hypoxic conditions within the tumor microenvironment are closely related to the advancement and metastasis of cancer (4). Hypoxia refers to a state where the oxygen concentration in tissues is below normal levels due to inadequate oxygen supply. In the tumor microenvironment, hypoxia arises from an imbalance between tumor cell proliferation (high oxygen consumption) with angiogenesis (sluggish oxygen delivery), showing an insufficient oxygen supply within the tumor (5, 6). Hypoxia can promote the infiltration and dissemination of neoplastic cells by activating a series of signaling pathways associated with tumor aggressiveness, such as the HIF-1α signaling pathway (7). HIF-1α is a key transcriptional factor in the cellular response to hypoxia, which can induce the production of VEGF and other angiogenic factors, thereby enhancing tumor angiogenesis and increasing the tumor’s invasiveness and metastatic capacity (8). HIF-1α is reported to counteract the effects of p53 during cancer progression (9). Increased expression of HIF-1α has been widely demonstrated a correlation with poor prognosis in gastric cancer patients (4, 10).

The tumor microenvironment constitutes an intricate network encompassing tumor cells, immune cells, fibroblasts, endothelial cells, and the surrounding extracellular matrix (11). Hypoxia can affect the biological behaviors of various cells within it. For example, hypoxic conditions can stimulate the activation of cancer-associated fibroblasts (CAFs), which are pivotal in the tumor microenvironment due to their ability to secrete cytokines and matrix metalloproteinases (MMPs), thereby enhancing tumor invasion and metastatic capabilities (12). Under hypoxic conditions, CAFs orchestrate an augmentation in tumor malignancy through diverse mechanisms such as extracellular matrix remodeling, immunological tolerance, metabolic restructuring, neovascularization, metastatic dissemination, and therapeutic resistance (13). Hypoxia stands as a prominent flaw within the tumor microenvironment, significantly impacting the efficacy of conventional radiotherapy and chemotherapy. It also plays a pivotal role in fostering malignant progression, encompassing the rapid and aggressive growth of primary tumors, their recurrence, and the dissemination of metastatic lesions (14).

Hypoxia also affects the function of immune cells. As well-known, T cells are key cell types in antitumor immune responses, but their activity is suppressed under hypoxic conditions (15, 16). Furthermore, immune cells present within the tumor microenvironment, namely tumor-infiltrating macrophages (TIMs), exert crucial functions in accelerating tumor expansion and dissemination to distant sites (17). They exert immunosuppressive effects by stimulating tumor angiogenesis, increasing the invasiveness and vascular invasion capabilities of tumor cells, also preventing NK cells or T cells from attacking tumor cells during cancer progression or recovery after chemotherapy (18). Hypoxia is closely related to resistance to tumor immunotherapy (19). Hypoxia can reduce the responsiveness of neoplastic cells to radiation therapy and pharmacological treatment because it can impair DNA damage repair capacity and disrupt drug metabolism and transport (20). Furthermore, hypoxia promotes the formation of Stem-like cells (21), which possess self-renewal capacity and the ability to differentiate into multiple cell types, serving as significant contributors to tumor relapse and therapeutic refractoriness (22).

In the context of STAD, recent studies on hypoxia have concentrated on the construction of hypoxia-related prognostic signature in predicting the clinical outcome (23, 24), along with the effect of hypoxia on the cellular behaviors (25, 26). Research has demonstrated that the expression levels of genes linked to hypoxia are intimately tied to the stage, grade, and clinical outcome of gastric adenocarcinoma (27). Moreover, hypoxia can serve as an autonomous prognostic indicator for gastric adenocarcinoma, with patients exhibiting high levels of hypoxia having a poorer prognosis (28). Given the role of hypoxia in the advancement of gastric adenocarcinoma, in-depth research into the molecular mechanisms of hypoxia, the exploration of hypoxia-related biomarkers, and the development of targeted therapeutic strategies against hypoxia are of considerable importance in the improvement of clinical outcomes for those with gastric adenocarcinoma. Therefore, we comprehensively utilize molecular biology, genomics, and other methodology to deeply study the influence of hypoxia in STAD and establish a hypoxia-related gene-based prognostic model, intending to offer innovative approaches and techniques for diagnosis and prognosis of STAD.

## 2 Methods

### 2.1 Collection and handling of transcriptomic information

In this study, RNA expression profiles and medical records from gastric cancer patients (n=368) were retrieved from TCGA database for modeling. To validate the model’s stability and accuracy, the GSE15460 (n=248) dataset from the GEO database, was employed as a validation set. All data underwent TPM (Transcripts Per Million) format conversion followed by log2 transformation to facilitate subsequent analysis. Above data was normalized by the sva and limma packages of R software (version 4.1.3).

### 2.2 Gathering and preparation of single-cell RNA sequencing data

The single-cell dataset was obtained from GSE183904, with 26 primary gastric cancer samples selected. We conducted the single-cell data analysis utilizing the Seurat package in the R. Quality control criteria included mitochondrial content below 20%, hemocyte content below 3%, and UMI counts and gene counts ranging from 200-20,000 and 200-6,000, respectively. Normalization of the data, identification of highly variable genes (amounting to 2000), and transformation of the data (to mitigate the influence of the cell cycle by regressing out “S.Score” and “G2M.Score” parameters) were executed utilizing the NormalizeData, FindVariableFeatures, and ScaleData functions from the Seurat package, respectively. To rectify batch effects, Harmony was employed. Subsequent steps involved dimensionality reduction techniques, encompassing UMAP, TSNE, and the Louvain clustering algorithm, all derived from the Seurat package. Differential gene expression analysis between clusters or between cell types was conducted using the FindAllMarkers function, with thresholds set at a p-value less than 0.05, a log2 fold change greater than 0.25, and an expression proportion exceeding 0.1.

### 2.3 Identifying hypoxic and non-hypoxic cells

CHPF (available at github.com/yihan1221/CHPF), an open-source software, was utilized for predicting cellular hypoxic conditions by integrating single-cell transcriptomic profiles with hypoxia-induced gene clusters. According to hypoxia status, cells were divided into hypoxic cells (H-group) and non-hypoxic cells (N-group cells) and visualized in the UMAP plot.

### 2.4 Cell annotation analysis

Cell marker genes were determined for neoplastic cells, myeloid cells, fibroblasts, endothelial cells, MAST cells, B cells, T cells, and NK cells. Individual cluster analyses were performed on tumor cells to investigate the diversity within the tumor, with results depicted through UMAP and t-SNE plots, as well as bar graphs and heatmaps.

### 2.5 WGCNA and enrichment evaluation

WGCNA package was used to examine gene modules correlated with the H-group cells, and performed gene enrichment analysis with the clusterProfiler package, taking advantage of the GO-BP and KEGG databases. The enrichment outcomes for both the H-group and N-group cells were graphically represented using the EnrichmentMap and AutoAnnotate plugins within the Cytoscape platform.

### 2.6 Analysis of cell-cell communication

CellChat package was used to assess potential intercellular communication. The gene expression matrix, once normalized, was fed into the CellChat framework to establish a CellChat entity. Preliminary data handling encompassed the application of functions such as identifyLIHCerExpressedGenes, identifyLIHCerExpressedInteraction, and ProjectData. The likelihood of ligand-receptor interactions was deciphered through the execution of computeCommunProb, filterCommunication, and computeCommunProbPathway utilities, culminating in the assembly of a network mapping cellular communications utilizing the aggregateNet function.

### 2.7 Single-cell CNV analysis

Employing the infercnv package, we estimated the copy number variations within the tumor cells, benchmarking against endothelial cells as a comparative baseline. For every tumor cell, a CNV score was derived to quantify these genetic alterations.

### 2.8 Single-cell transcription factor assessment

The SCENIC package was employed to predict transcription factors in H1 and N1 cell populations, with GRNboost2 software used for gene co-expression analysis to construct gene regulatory networks. Important nodes in the network were assessed by degree, and the top 1% of genes or transcription factors were chosen for in-depth examination.

### 2.9 Development of a predictive outcome model

Transcription factors only derived from the H1 cell population were analyzed. Initially, a univariate Cox regression analysis was conducted to filter out genes associated with survival outcomes. The LassoCox algorithm was employed for modeling to establish a prognostic model and calculate risk scores. The cutoff value was established as the median, categorizing patients into high-risk and low-risk groups accordingly.

### 2.10 Validation of the predictive outcome model

This study used the GEPIA2 (Gene Expression Profiling Interactive Analysis 2) platform to analyze the expression of genes in the model in STAD. GEPIA2 is an online tool based on the TCGA and GTEx (Genotype-Tissue Expression) databases, providing functions for differential gene expression analysis between cancer and normal tissues, as well as clinical data correlation analysis. The significance level was set at P < 0.05.

### 2.11 RNA extraction and quantitative real-time PCR analysis

Total RNA was extracted from tissues and cell lines using Trizol reagent (Takara Inc., Dalian, P.R. China). Subsequently, cDNA was synthesized using the PrimeScript RT Reagent Kit (Takara Inc., Dalian, P.R. China) with 500 ng of total RNA. Quantitative real-time PCR (qRT-PCR) was performed using SYBR Premix Ex Taq™ (Takara Inc., Dalian, P.R. China) on a CFX96 Thermal Cycler Dice™ Real-Time PCR System (Bio-Rad Laboratories, Inc., CA). All samples were run in triplicates, and the mRNA expression levels of the target genes were normalized to 18S rRNA cDNA expression levels. The primer sequences are listed in **Supplementary Table 1**. GES-1, AGS, BGC823, and MGC803 cell lines were purchased from the ATCC (American Type Culture Collection) cell bank.

### 2.12 Statistical analysis

All phases of data manipulation, statistical calculations, and data visualization were carried out using R software (version 4.1.3). The relationship between two continuous variables was evaluated by determining the Pearson correlation coefficient. For comparing categorical variables, a Chi-square test was used, whereas for continuous variables, either the Wilcoxon rank-sum test or the t-test was chosen based on the data’s characteristics. Cox regression analysis and Kaplan-Meier survival analysis were conducted using the survival package in R.

## 3 Results

### 3.1 Single-cell atlas of STAD

**Figure 1:**
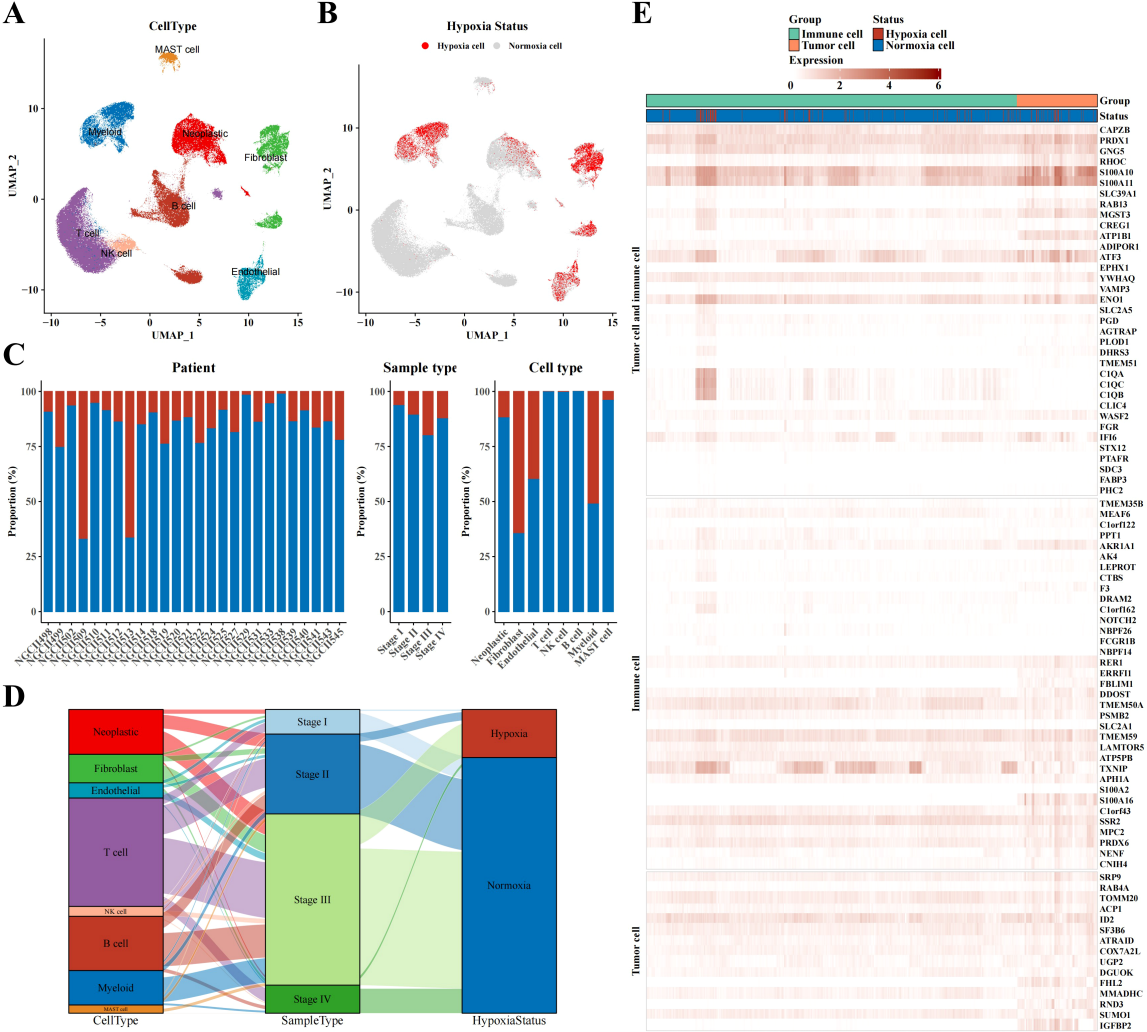
Single-cell atlas results of STAD. **(A)** UMAP plot visualizing annotated cell type from single-cell data. **(B)** UMAP plot identifying hypoxia status of different cell types using CHPF software. **(C)** Bar chart representing the proportion of hypoxia status across samples, tissue types, and cell types. **(D)** Sankey diagram correlating cell type, sample type, and hypoxia status. **(E)** Heatmap depicting marker expression in tumor and immune cells based on hypoxia status.

The UMAP plot demonstrated different cell types in STAD tissues, including neoplastic cells, myeloid cells, fibroblasts, endothelial cells, MAST cells, B cells, T cells, and NK cells (**Figure 1A**). Markers were identified in neoplastic cells (CDH1, EPCAM, KRT18, KRT19), fibroblasts (SLRR1B, CD90, COL1A1, COL1A2), endothelial cells (CD31, CLDN2, VEGFR-1, RAMP2), T cells (CD3D/E/G, IMD7), NK cells (GIG1, NKG5, CD56, CD94), B cells (CD79A, AGM1, IgG3, IGHA2), myeloid cells (AMYLD5, SCARA2, CD16, CD68), and mast cells (CD117, ATOPY, DCML). We conducted the CHPF software to classify cells into two categories, revealing that most of neoplastic cells, fibroblasts, endothelial cells, and myeloid cells were hypoxic, whereas most of mast cells, NK T cells, and B cells were non-hypoxic (**Figure 1B**). Further analysis of the ratio of hypoxic cells across disparate samples and tissue types showed a significant increase in the proportion of hypoxic cells in stage III STAD tissues; fibroblasts had the highest proportion of hypoxic cells among all cell subpopulations, followed by endothelial cells, myeloid cells, and neoplastic cells (**Figure 1C**). A Sankey diagram revealed the associations between cell type, sample type, and hypoxic status, indicating that hypoxic cells were concentrated mainly in key cell subpopulations of stage III STAD, such as neoplastic cells, fibroblasts, endothelial cells, and myeloid cells (**Figure 1D**). The heatmap displayed the expression of hypoxia-related marker genes in neoplastic and immune cells, demonstrating that immune cells also exhibited a clear hypoxic state (**Figure 1E**).

### 3.2 Analysis of immune cell subpopulations

**Figure 2:**
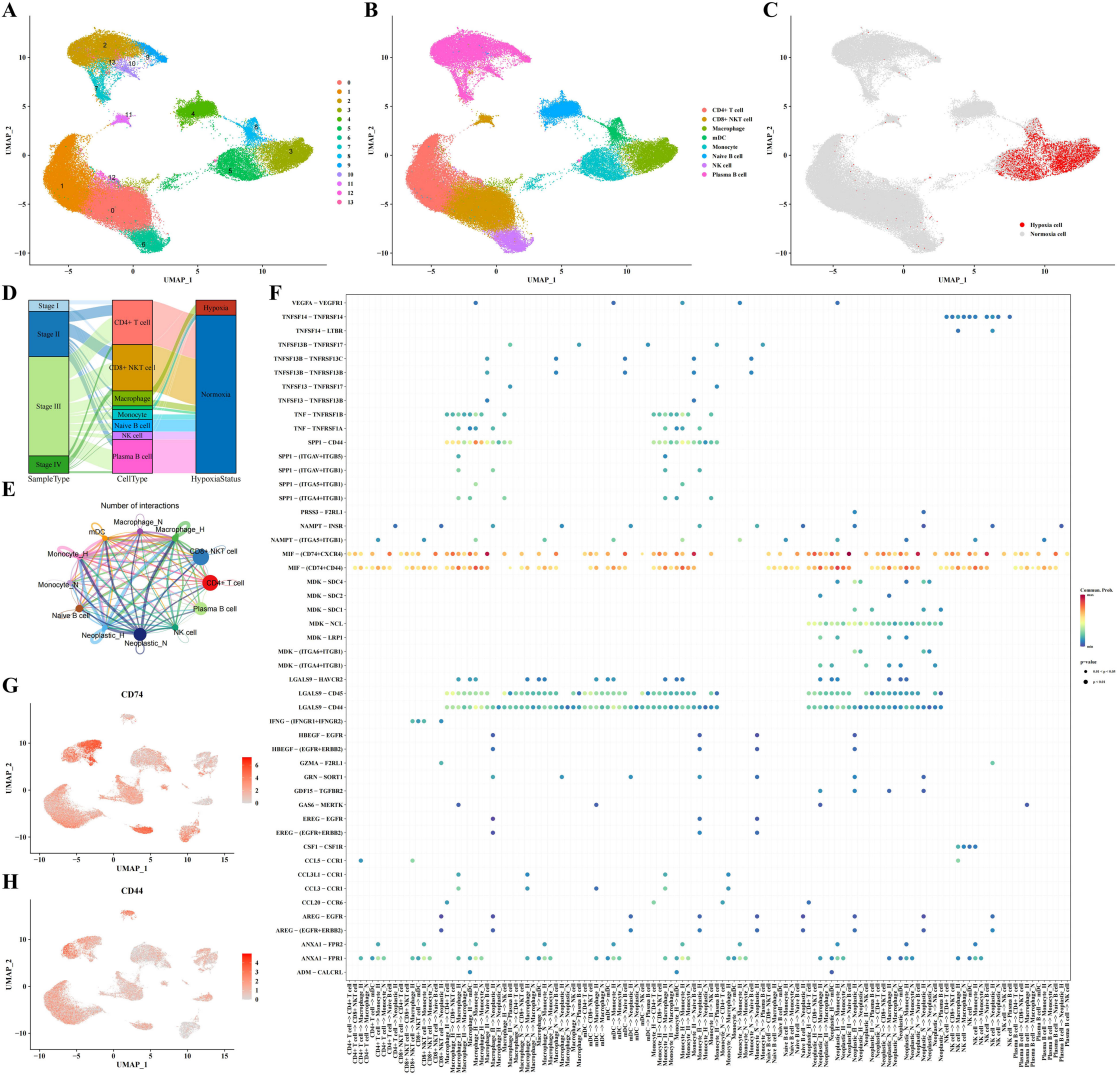
Immune cell subpopulation analysis results. **(A-C)** Clustering, cell type annotation, and hypoxia status UMAP plots of immune cell subpopulations. **(D)** Sankey diagram correlating cell type, sample type, and hypoxia status. **(E, F)** Analysis results of communication between immune cells and tumor cells. **(G)** UMAP plot indicating expression profile of CD74 based on single-cell data. **(H)** UMAP plot indicating expression profile of CD44 based on single-cell data.

In this part, we delved into the characteristics of immune cells in a hypoxic microenvironment. Dimensionality reduction clustering analysis depicted the distribution of immune cell subpopulations and distinguished hypoxic from non-hypoxic states, revealing that hypoxic cells were concentrated mainly in macrophages, myeloid dendritic cells (mDCs), and monocytes (**Figures 2A-C**). Sankey diagram analysis showed that these hypoxic cells were primarily derived from STAD stages II and III (**Figure 2D**). Using CellChat software, we analyzed the communication between immune cells and neoplastic cells, revealing a potential cell-cell communication network and presenting communication and receptor-ligand interaction plots (**Figure 2E**). As shown in **Figure 2F**, we found high communication activity between CD74 and CD44, so that we visualized the expression levels of CD74 and CD44 in **Figures 2G, H**. In addition, we depicted the cell communication pathways in diverse immune and tumor cells including incoming and outgoing patterns, showing the importance of MIF signaling pathway (**Supplementary Figure 1A**). Correspondingly, the heatmap of the MIF signaling pathway communication between different cell types was presented in **Supplementary Figure 1B**.

### 3.3 Analysis of neoplastic cell subpopulations

**Figure 3:**
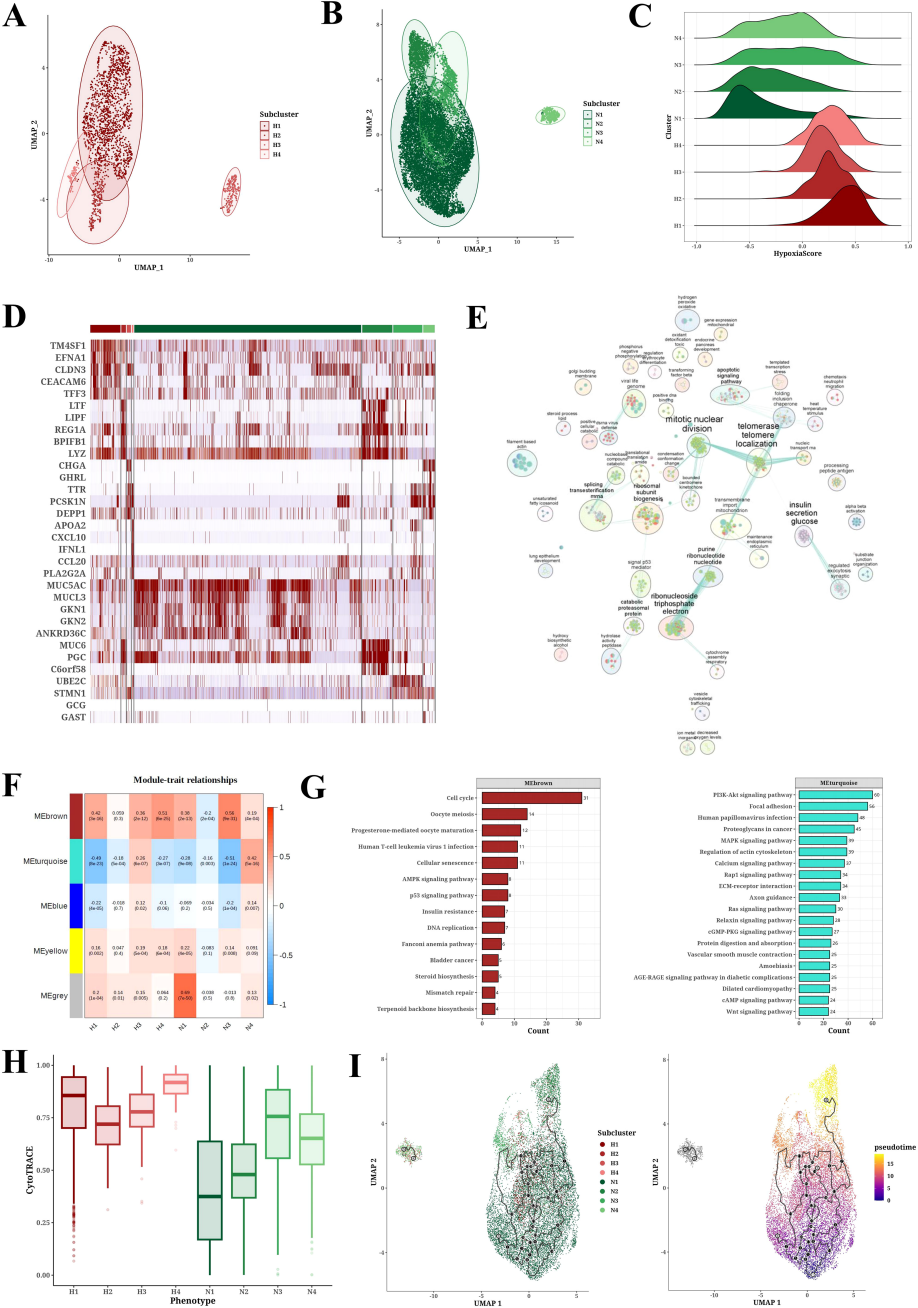
Tumor cell subpopulation analysis results. **(A, B)** UMAP plots of PCA analysis of hypoxic (H1-H4) and non-hypoxic (N1-N4) cell subclusters. **(C)** Ridge plot of hypoxia scores among hypoxic (H1-H4) and non-hypoxic (N1-N4) cell populations. **(D)** Heatmap of marker expression in each cell population. **(E)** GO-BP enrichment analysis results for each cell population. **(F)** Module-trait relationships between gene modules and cell populations through WGCNA analysis. **(G)** KEGG enrichment analysis results for gene modules brown and turquoise. **(H)** CytoTRACE analysis results for each cell population. **(I)** Pseudotime analysis results of cell populations (H1-H4, N1-N4) derived from Monocle3.

Cluster analysis of neoplastic cells based on their hypoxic status identified four hypoxic subpopulations (H1-H4) and four non-hypoxic subpopulations (N1-N4) (**Figures 3A, B**). Further hypoxia scores indicated that the H1 subpopulation had the highest degree of hypoxia (**Figure 3C**). Heatmap analysis showed the marker genes of each cell cluster, with genes such as TM4SF1, EFNA1, CLDN3, and CEACAM6 significantly upregulated in hypoxic cell subpopulations (**Figure 3D**). GO-BP enrichment analysis and the application of Cytoscape software revealed the functional enrichment of hypoxic subpopulation marker genes, involving biological processes such as mitotic nuclear division, telomerase telomere localization, insulin secretion glucose, and ribonucleoside triphosphate electron (**Figure 3E**). WGCNA analysis identified key gene modules associated with each subpopulation, such as the high correlations between the H2 subpopulation and the red module and between the H3 subpopulation and the yellow module (**Figure 3F**). WGCNA analysis revealed brown module was positively correlated to hypoxic subpopulations and turquoise module was negatively correlated to hypoxic subpopulations (**Figure 3F**). GO-BP enrichment analysis of these gene modules revealed their involvement in biological processes of cell cycle, progesterone-mediated oocyte maturation, and oocyte meiosis, while KEGG results suggested their involvement in PI3K-Akt signaling pathway, focal adhesion, human papillomavirus infection, proteoglycans in cancer, MAPK signaling pathway, and regulation of actin cytoskeleton (**Figure 3G**). CytoTRACE analysis showed that the H4 subpopulation appeared the peak differentiation level compared to non-hypoxic subpopulations (N1-N4) (**Figure 3H**). Pseudotime analysis using monocle3 software revealed that neoplastic cells with high hypoxia scores were located in the middle of the pseudotime trajectory (**Figure 3I**). **Supplementary Figure 2** provides UMAP plots of cell subpopulation distribution and hypoxia score enrichment by Pseudotime analysis, along with the distribution of hypoxia score in different H1-H4 subclusters.

### 3.4 Analysis of tumor-related pathways, CNV, and transcription factors

**Figure 4:**
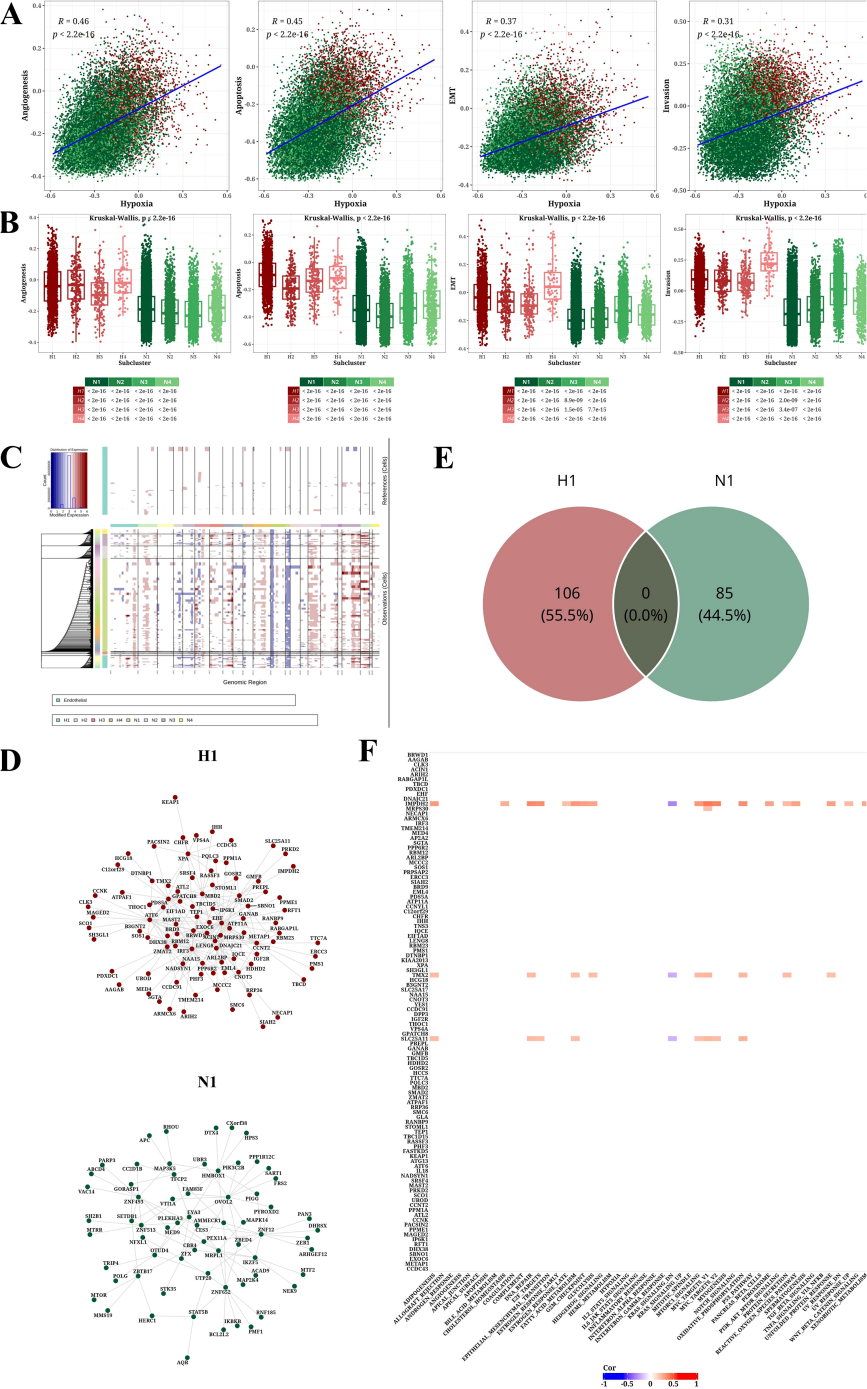
Analysis results of tumor-related pathways, CNV, and transcription factors. **(A)** Scatter plot showing correlations between hypoxia score and four other signature scores (Angiogenesis, Apoptosis, EMT, and Invasion). **(B)** Box plot illustrating differences in four signature scores (Angiogenesis, Apoptosis, EMT, and Invasion) across cell populations. **(C)** CNV prediction results using InferCNV software with endothelial cells as normal reference. **(D)** Network diagram of top 1% transcription factors/genes for H1 and N1. **(E)** Venn diagram of top 1% transcription factors/genes for H1 and N1. **(F)** Heatmap of correlation between h1-specific transcription factors/genes in and hallmark pathways (|Cor|>0.2 and P<0.05).

To investigate the link between hypoxia and the aggressiveness of STAD, we sourced signature genes pertinent to hypoxia, invasion, apoptosis, angiogenesis, and EMT from the CancerSEA database and computed activity scores for each cellular subpopulation employing GSVA analysis (**Supplementary Figure 3**). Correlation analysis demonstrated the positive associations of hypoxia score with Angiogenesis, Apoptosis, EMT, and Invasion scores (**Figure 4A**). In **Figure 4B**, it revealed that the Angiogenesis, Apoptosis, EMT, and Invasion scores of hypoxic subpopulation exhibited significantly higher than non-hypoxic subpopulation. CNV analysis unveiled CNV scores among diverse tumor subpopulations (**Supplementary Figure 4**), and the CNV status of cancer cells was evaluated using endothelial cells as a benchmark (**Figure 4C**).

Transcription factor analysis further identified in the H1 and N1 subpopulations and extracted the top 1% of associated nodes (**Figure 4D**). We identified 106 and 85 key transcription factors in the H1 and N1 subpopulations, respectively. The overlapped transcription factors of H1 and N1 subpopulations were zero, indicating the specificity of identification in **Figure 4E**. Correlation analysis between 106 H1-specific transcription factors and Hallmark pathways was conducted, presenting a heatmap of correlations with |Cor|>0.2 and P<0.05 (**Figure 4F**).

### 3.5 Construction of the prognostic model by LASSO

**Figure 5:**
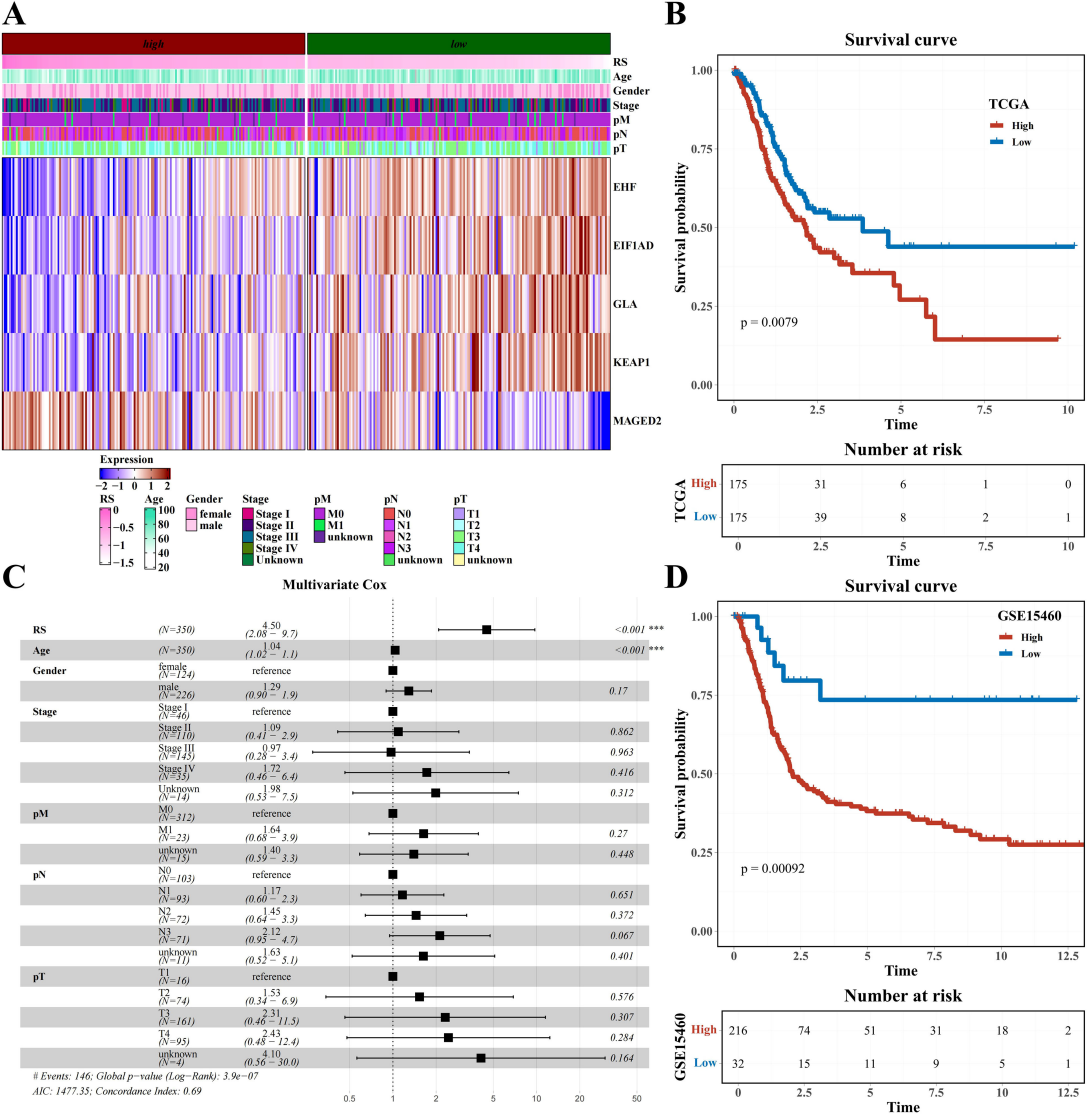
Prognostic model construction based on H1-specific transcription factors/genes **(A)** Heatmap of expression of modeling genes EHF, E1F1AD, GLA, KEAT1, and MAGED2 (including clinical indicators of age, gender, stage, pM, pN, and pT). **(B)** Survival analysis results of risk groups from TCGA Dataset. **(C)** Forest plot of multivariate cox analysis including risk score and clinical indicators age, gender, stage, pM, pN, and pT. **(D)** Survival analysis results of risk groups from GSE15460 Dataset.

The LassoCox algorithm was employed to refine the construction of the prognostic model (**Supplementary Figures 5A, B**), and patients were stratified into high- and low-risk categories based on the median risk score. Ultimately, five transcription factors—EHF, EIF1AD, GLA, KEAPI, and MAGED2—were identified for constructing the prognostic model (**Supplementary Figures 5C, D**; **Figure 5A**). The Kaplan-Meier survival curve revealed a significantly reduced overall survival (OS) rate in the high-risk group compared to the low-risk group within the TCGA dataset (P=0.0079) (**Figure 5B**). Multivariate Cox analysis further confirmed that this model served as an independent predictor of prognosis for STAD patients, with an odds ratio (OR) of 4.50 (95% CI: 2.08-9.70), P<0.001 (**Figure 5C**). The prognostic value of the model was validated in GSE15460, demonstrating a significantly lower survival rate in the high-risk group versus the low-risk group of STAD patients (P=0.00092) (**Figure 5D**).

### 3.6 Drug analysis

**Figure 6:**
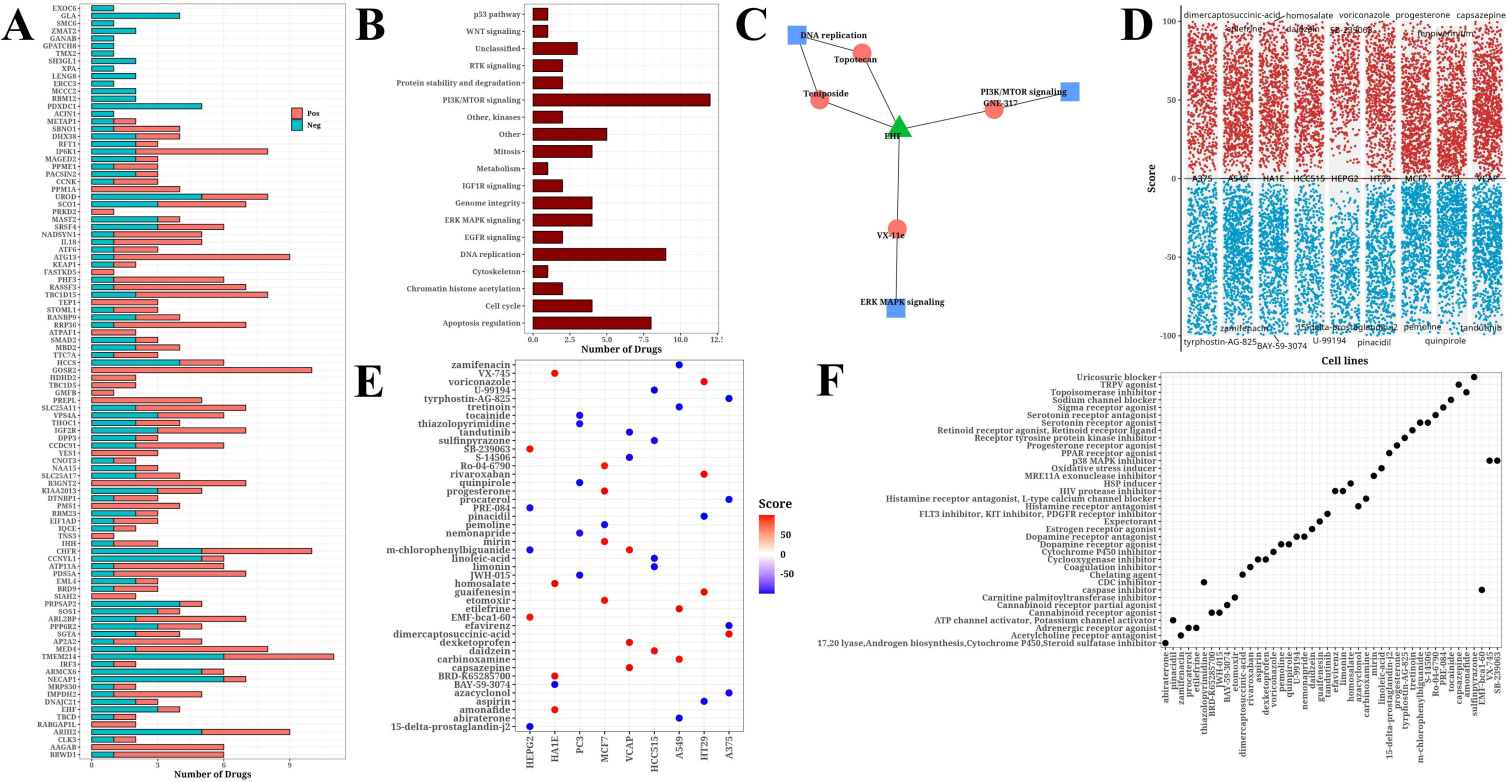
Drug analysis results. **(A)** Bar chart of significant correlation between H1-specific transcription factors and drugs. **(B)** Bar chart of target pathways for drugs. **(C)** Network diagram of model gene EHF, drugs, and target pathways. **(D)** Volcano plot from CMap online analysis showing compound scores across cell lines. **(E)** Bubble plot of Top 5 compounds (|score|) selected from each cell line. **(F)** Bubble plot of target pathways corresponding to top 5 compounds (|score|) selected from each cell line.

Based on information from the GDSCv2 database, we calculated the correlation between H1-specific transcription factors and drugs with |Cor|>0.3 and P<0.05, and presented a bar chart (**Figure 6A**). These drugs target multiple crucial tumor development and progression processes, including PI3K/mTOR signaling, DNA replication, and apoptosis regulation (**Figure 6B**). The transcription factor EHF was found to be significantly associated with multiple drugs targeting different biological pathways, including Topotecan and Teniposide acting on DNA replication, GNE317 acting on PI3K/mTOR signaling, and VX-11e acting on ERK/MAPK signaling (**Figure 6C**). Using online analysis from the CMap database (QUERY [clue.io]), we submitted H1-specific transcription factors as upregulated genes and N1-specific transcription factors as downregulated genes for analysis. The resulting volcano plot displayed compound scores across various cell lines (**Figure 6D**). Further analysis of the top 5 compounds ranked by |Score| in each cell line yielded a total of 44 compounds (**Figure 6E**). Finally, statistical analysis of the pathways targeted by these compounds was conducted (**Figure 6F**), identifying potential candidates for future hypoxia-targeted therapies.

### 3.7 *In-vitro* validation of expression of modeling genes

**Figure 7:**
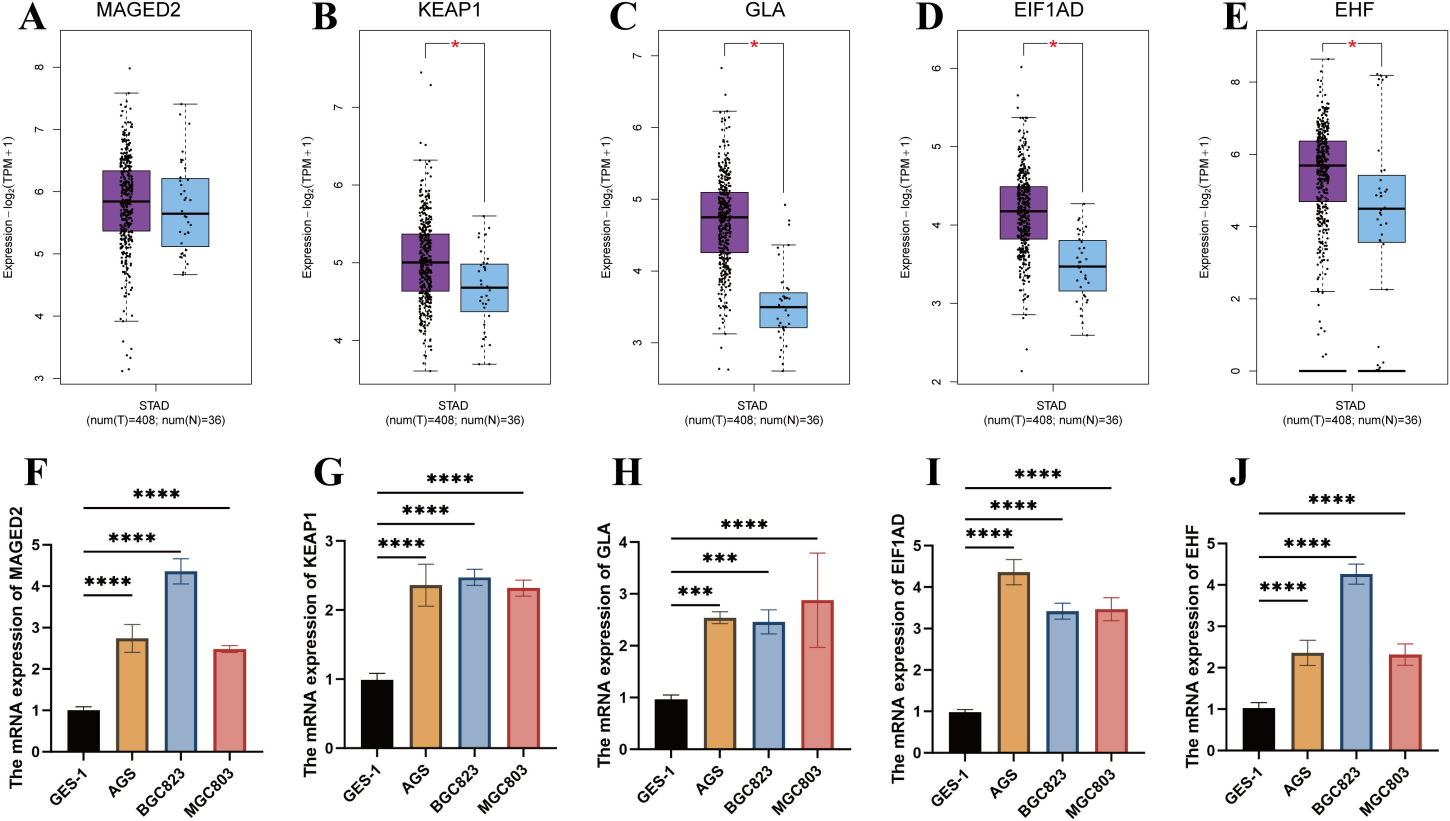
Validation of gene expression in the prognostic model. **(A-E)** Expression analysis of MAGED2, KEAP1, GLA, EIF1AD, and EHF between tumor and normal tissues in STAD using GEPIA2. **(F-J)** Expression analysis of MAGED2, KEAP1, GLA, EIF1AD, and EHF between AGS, BGC823, MGC803 cell lines and the normal control group (GSE-1).

The expression levels of MAGED2, KEAP1, GLA, EIF1AD, and EHF genes were analyzed in the TCGA-STAD dataset using the GEPIA2 platform. Except for MAGED2, the expression levels of the remaining four genes were significantly higher in tumor tissues compared to normal tissues (P < 0.05) (**Figures 7A-E**). Furthermore, we compared the expression levels of these genes in commonly used gastric cancer cell lines AGS, BGC823, MGC803, and the normal control group GES-1. The results showed that the expression of all five genes was significantly higher in gastric cancer cell lines compared to normal tissues and the control group (P < 0.001) (**Figures 7F-J**). These findings further support the potential roles of MAGED2, KEAP1, GLA, EIF1AD, and EHF genes in the occurrence and progression of STAD.

## 4 Discussion

This research explores the prognostic signature of MAGED2, KEAP1, GLA, EIF1AD, and EHF genes associated with hypoxia in gastric cancer through analyzing transcriptome information and scRNA-seq data, and indicates the clinical relevance of hypoxia during gastric cancer progression.

Our findings reveal that hypoxia run a pivotal role in gastric cancer. Cells within gastric cancer tissues can be categorized into hypoxic and non-hypoxic groups, with neoplastic cells, fibroblasts, endothelial cells, and myeloid cells predominantly being hypoxic. This finding is consistent with prior research findings, indicating that the prevalence of hypoxic cells within the tumor microenvironment is intimately tied to the aggressiveness of the tumor and its clinical outcome (29, 30). Furthermore, we found a significant increment in the proportion of hypoxic cells in stage III gastric cancer tissues, potentially linked to rapid tumor proliferation and inadequate angiogenesis (31). Hypoxia influences both the biological behavior of neoplastic cells and the other cell types present in the tumor microenvironment (32, 33). Hypoxia can promote the activation of cancer-associated fibroblasts (CAFs) (34), which facilitate tumor invasion and metastasis by secreting cytokines and matrix metalloproteinases (MMPs) (35). Furthermore, hypoxia alters the functioning of immune cells by suppressing T-cell activity, consequently impairing the immune response against the tumor (36, 37). These discoveries underscore the intricate role of hypoxia within the tumor microenvironment and its diverse effects on the progression of gastric cancer. Maintaining a stable oxygen environment is important to provide the oxygen needed for oxidative phosphorylation and to defend cells against oxidative stress (38). Hypoxia induces a metabolic shift in tumor cells, transitioning from oxidative phosphorylation to anaerobic glycolysis (39), which is characterized by reduced energy efficiency and heightened glucose consumption. In hypoxic environments, cancer cells exhibit weaker antioxidant defenses, rendering them more susceptible to the direct damaging effects of ROS (40). Additionally, ROS can bolster antitumor immune responses by promoting mutations and the generation of immunogenic neoantigens (41). Significant increases in ROS levels have been detected in gastric cancer patients, with these high levels causing oxidative stress that may damage the gastric mucosa and contribute to cancer progression (42). Shedding light on the hypoxia-related development of gastric cancer and finding effective biomarkers is vital for improving the diagnosis, prevention, and management of gastric cancer.

Through cluster analysis of neoplastic cells based on their hypoxic status, we identified four hypoxic and four non-hypoxic subpopulations. Further hypoxia scoring revealed that the H1 subpopulation had the highest degree of hypoxia. The expression pattern of hypoxia-related genes in this subpopulation is closely related to tumor aggressiveness and prognosis. Additionally, WGCNA analysis and GO-BP enrichment analysis unveiled the functional enrichment of marker genes in hypoxic subpopulations, involving multiple biological processes and signaling pathways such as cell cycle, angiogenesis, PI3K-Akt and MAPK signaling pathways.

The connection between hypoxia and tumor resistance to treatment has been a key area of investigation. Our research suggests that hypoxia may diminish the responsiveness of tumor cells to radiotherapy and chemotherapy, potentially due to a reduced capacity for DNA damage repair and impairments in drug metabolism and transport induced by hypoxic conditions. In response to low oxygen levels, tumor cells adapt by activating HIF-1 and its downstream target genes, such as BNip3 and BNip3L (43). Under hypoxic conditions, the antioxidant defenses of tumor cells weaken, making them more vulnerable to the direct detrimental effects of ROS. Oxidative stress can trigger lipid peroxidation, endoplasmic reticulum stress, and dysfunction of Tregs, all of which contribute to immune dysregulation (44). Furthermore, hypoxia upregulates PD-L1 expression via HIF-1α, leading to the suppression of T-cell activation (45, 46). Blocking HIF-1α may aid in modulating the function and differentiation of myeloid-derived suppressor cells (MDSCs), thereby potentiating the antitumor immune response (46).

The biological traits of tumor cells can be influenced by gastric cancer cells in their microenvironment, which affect the expression of certain transcription factors and genes linked to tumors to adapt to hypoxia (47). Hypoxia-associated transcription factors EHF, EIF1AD, GLA, KEAPI, and MAGED2 were screened and applied for developing an excellent prognostic model for STAD by this study. Its prognostic performance was demonstrated by the K-M curve, indicating the worse OS in high-risk patients than in low-risk patients. Differential expression profile of genes associated with hypoxia can function as a standalone indicator of prognosis in gastric cancer, offering novel molecular targets for personalized therapeutic strategies in gastric cancer. Among the screened transcription factors, EHF can enhance or inhibit the expression of subsequent gene targets by forming transcriptional complexes alone or with other effector molecules, participating in processes such as cell proliferation, differentiation, apoptosis, and senescence (48, 49). *In vitro* experiments have confirmed that knocking down EHF in gastric cancer cells significantly reduces their clonal formation ability, invasion, and migration capacity, leads to cell cycle arrest, decreased proliferation, and increased apoptosis (50). Mechanistically, EHF binds to the HER2 promoter region to promote its transcription and activates the downstream pathways of MAPK/Erk and PI3K/AKT to promotes gastric tumorigenesis (51). Previous study indicated overactivation of the RAS/MAPK and PI3K/AKT/mTOR pathways results in the upregulation of HIF-1α (52), which is involved in the gastric cancer cell proliferation and invasion under hypoxic conditions. These findings indicated the potential coeffect of transcription factors EHF and HIF-1α on the progression of STAD, which needs more evidence to verify in the future.

The novelty of our research is as follows. Our study innovatively classifies the tumor cells into hypoxic cells (H1-H4) and non-hypoxic cells (N1-N4) based on single-cell sequencing data and determines the H1 subpopulation with the highest degree of hypoxia. H1-specific transcription factors were utilized to build a novel prognostic signature through LASSO algorithm for STAD. We validated *in vitro* that hypoxia-related model genes were highly expressed in tumor cells compared to normal cells to support the findings.

Despite providing in-depth insights into the character of hypoxia in STAD, this study has some limitations. Firstly, it primarily relies on bioinformatics analysis and requires further experimental validation to confirm the functions and clinical relevance of hypoxia-related genes. Secondly, the scope of this study is constrained by the sample size, and the accuracy and universality of the prognostic model need to be validated in larger samples. Finally, the dynamic changes and spatiotemporal heterogeneity of hypoxia in gastric cancer development require further *in-vivo* investigation.

## 5 Conclusion

A prognostic model was based on the hypoxia-associated transcription factors EHF, EIF1AD, GLA, KEAPI, and MAGED2, demonstrating remarkable efficacy in predicting the clinical outcomes of patients with STAD. These discoveries not only elevate our comprehension of the hypoxic influence in the progression of STAD but also illuminate novel molecular markers and targeted therapeutic avenues tailored for individual strategies.

## Data Availability

The original contributions presented in the study are included in the article/**Supplementary Material**. Further inquiries can be directed to the corresponding author/s.

## Supplementary material

Supplementary Figure 1 Cell Communication Analysis. **(A)** Heatmap of Cell Communication Results (Including Incoming and Outgoing patterns) for Single-Cell Data (Including Immune and Tumor Cells). **(B)** Heatmap of MIF Signaling Pathway Communication Between Different Cell Types.

Supplementary Figure 2
**(A)** Pseudotime Analysis Results of Cell Populations Derived from Monocle3. **(B)** Pseudotime Analysis Results of Hypoxia Score Derived from Monocle3. **(C)** Kruskal-Wallis Analysis Results of Hypoxia Score in Different H1-H4 Subclusters.

Supplementary Figure 3 Heatmap of GSVA Scores for Hallmark Pathways Across Hypoxic and Non-Hypoxic Tumor Subpopulations.

Supplementary Figure 4 Box Plot of CNV Scores for Hypoxic and Non-Hypoxic Tumor Subpopulations.

Supplementary Figure 5
**(A, B)** Modeling Results Using Lasso regression analysis. **(C)** Forest Plot of Univariate Cox Analysis for five Modeling Genes. **(D)** Bar Chart of Coefficients (Coef) for the Prognostic Model. * represents p < 0.05, ** represents p < 0.01, *** represents p < 0.001, and **** represents p < 0.0001.

Supplementary Table 1 qRT-PCR primers used in this study.
