# Supplementary figures and images for: Hypoxia-associated genes as predictors of outcomes in gastric cancer: a genomic approach
Source: Front Immunol. 2025 Mar 10;16:1553477. doi: 10.3389/fimmu.2025.1553477 (PMC11931070; doi:10.3389/fimmu.2025.1553477)

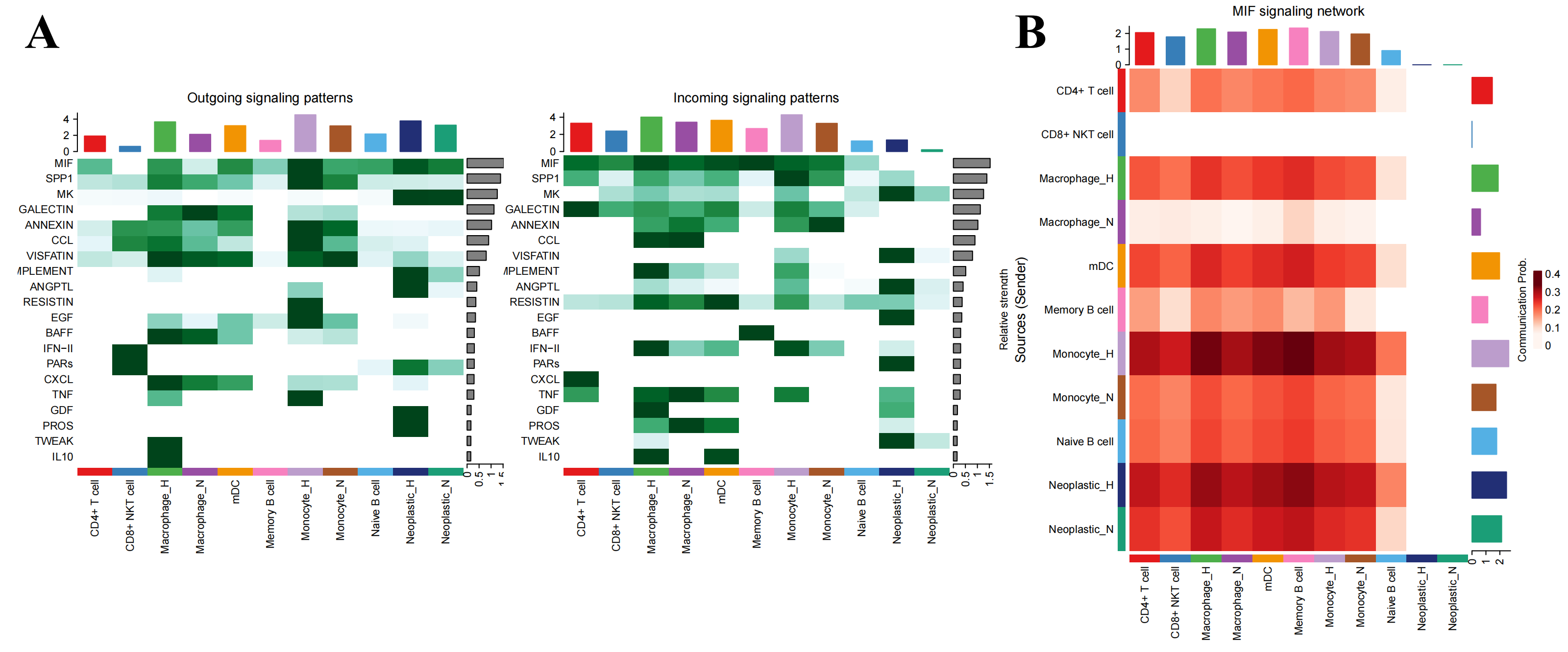

Supplement: Supplementary Figure 1 — Cell Communication Analysis. (A) Heatmap of Cell Communication Results (Including Incoming and Outgoing patterns) for Single-Cell Data (Including Immune and Tumor Cells). (B) Heatmap of MIF Signaling Pathway Communication Between Different Cell Types. [file Image1.tif]

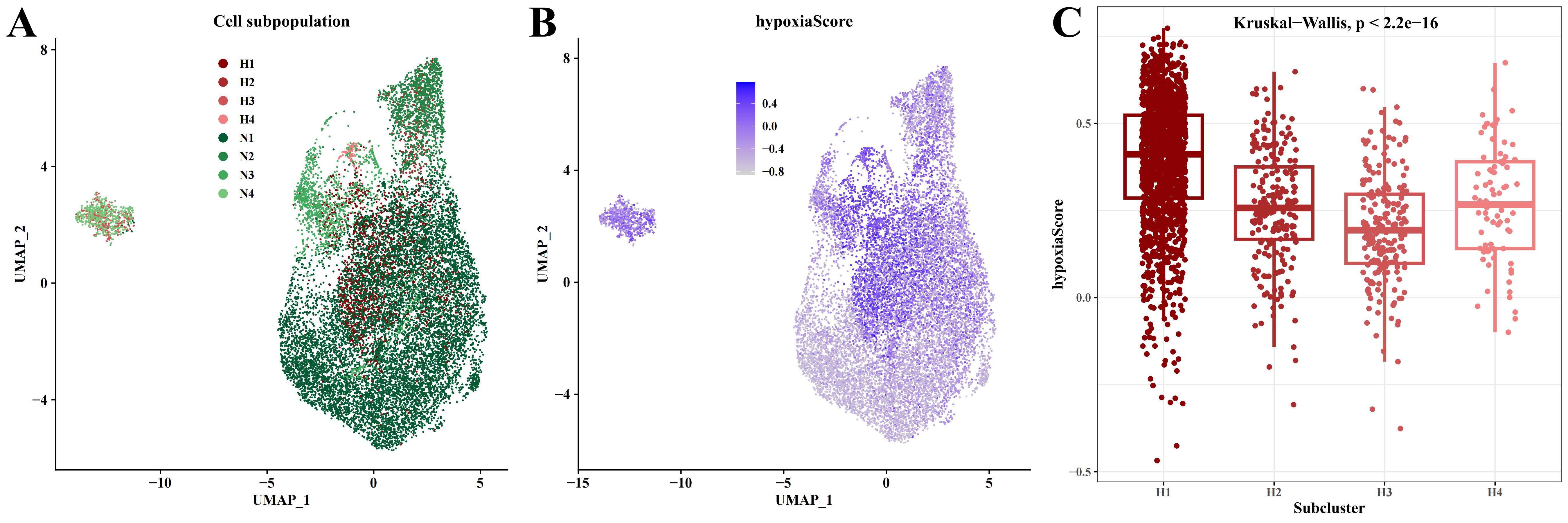

Supplement: Supplementary Figure 2 — (A) Pseudotime Analysis Results of Cell Populations Derived from Monocle3. (B) Pseudotime Analysis Results of Hypoxia Score Derived from Monocle3. (C) Kruskal-Wallis Analysis Results of Hypoxia Score in Different H1-H4 Subclusters. [file Image2.jpeg]

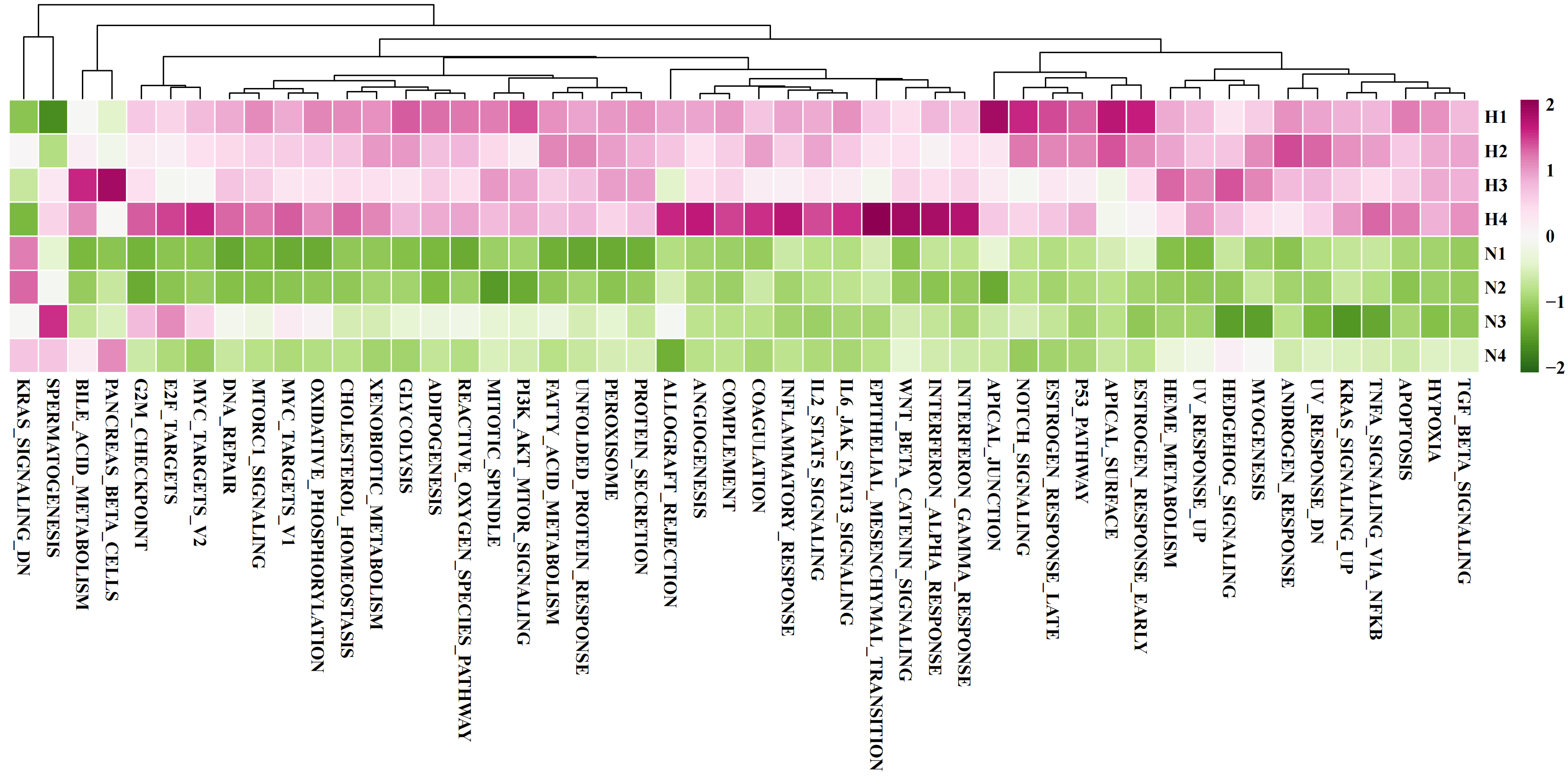

Supplement: Supplementary Figure 3 — Heatmap of GSVA Scores for Hallmark Pathways Across Hypoxic and Non-Hypoxic Tumor Subpopulations. [file Image3.tif]

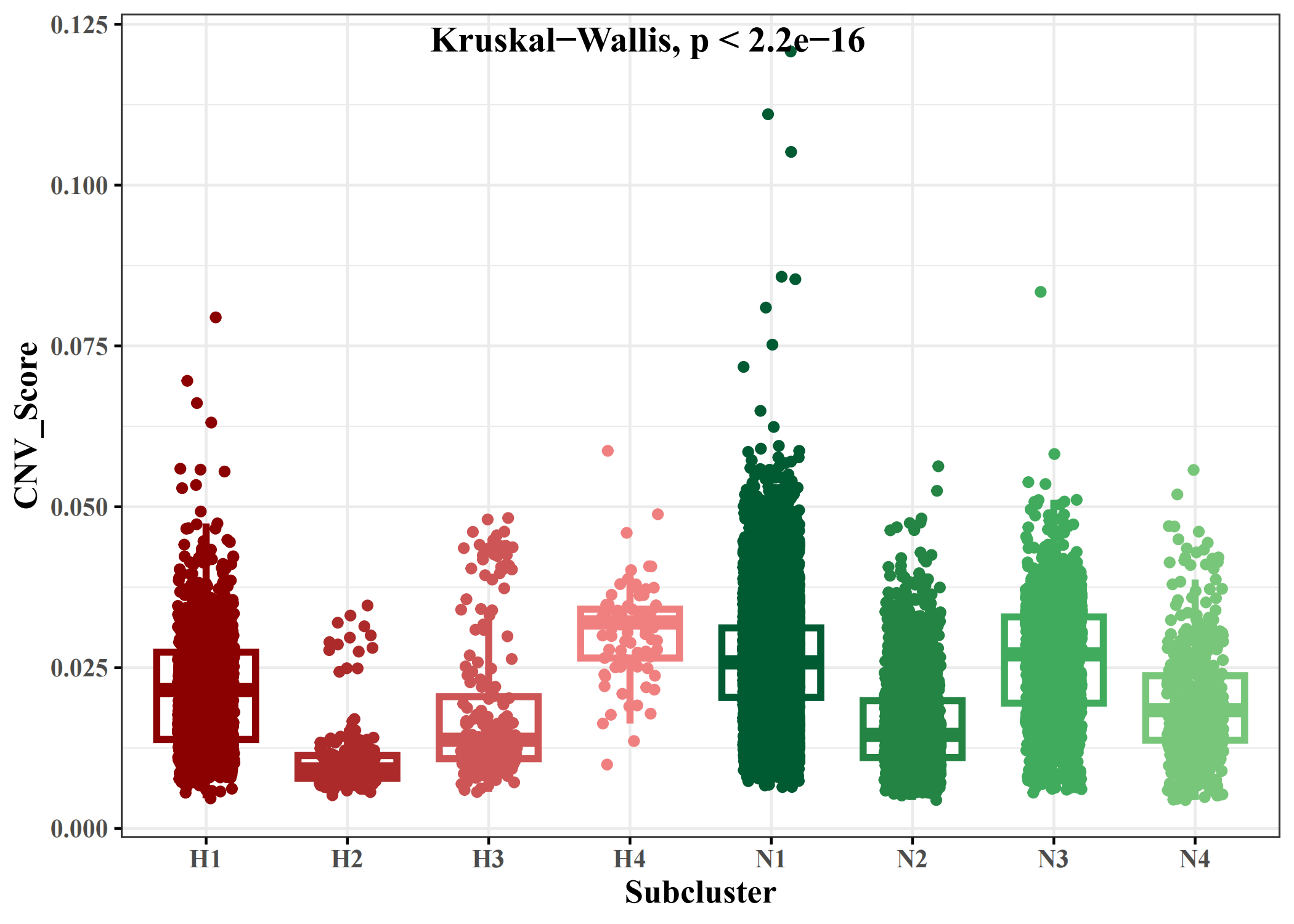

Supplement: Supplementary Figure 4 — Box Plot of CNV Scores for Hypoxic and Non-Hypoxic Tumor Subpopulations. [file Image4.tif]

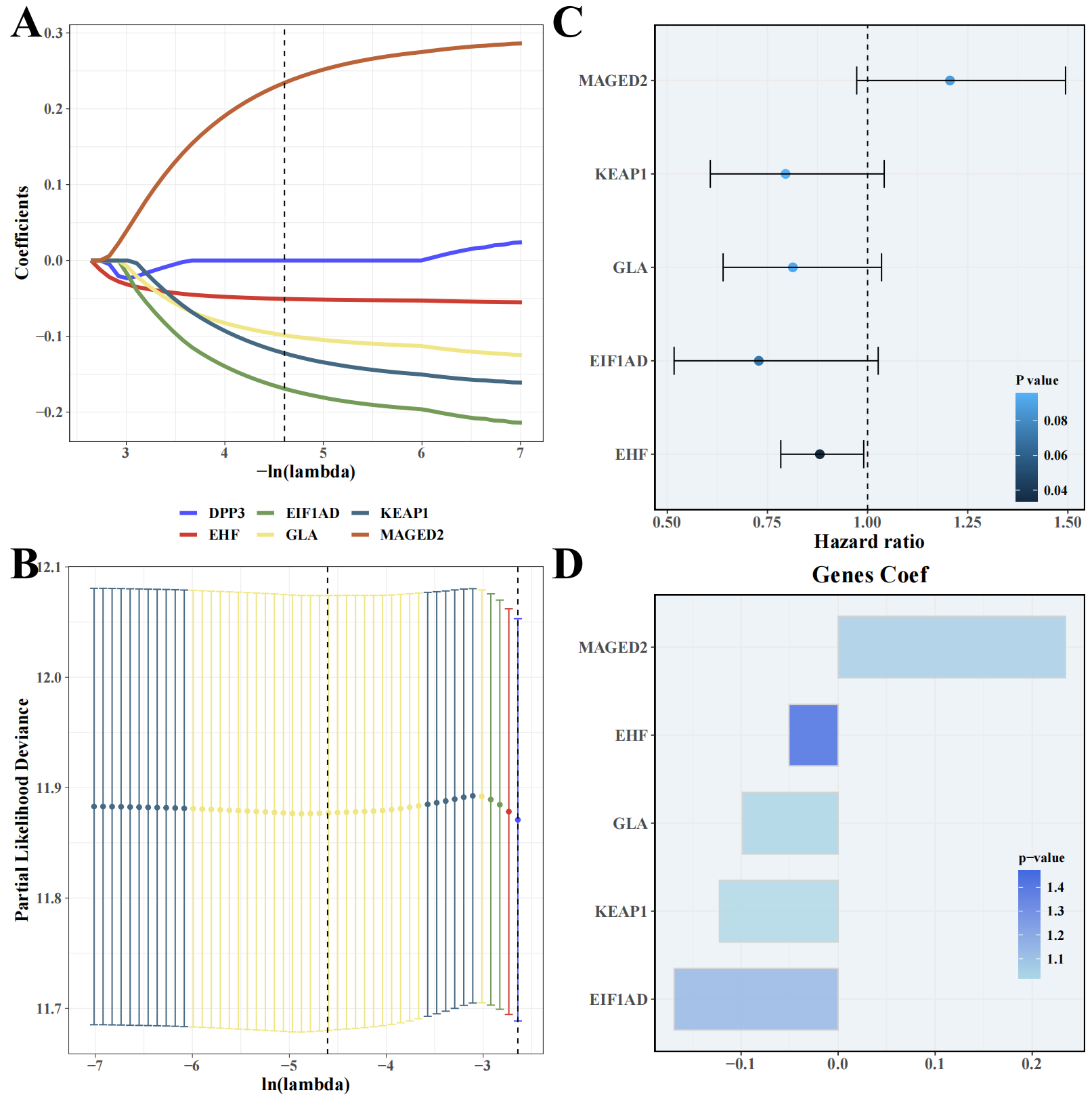

Supplement: Supplementary Figure 5 — (A, B) Modeling Results Using Lasso regression analysis. (C) Forest Plot of Univariate Cox Analysis for five Modeling Genes. (D) Bar Chart of Coefficients (Coef) for the Prognostic Model. * represents p < 0.05, ** represents p < 0.01, *** represents p < 0.001, and **** represents p < 0.0001. [file Image5.tif]
