# Supplementary material for: Hypoxia-associated genes as predictors of outcomes in gastric cancer: a genomic approach
Source: Front Immunol. 2025 Mar 10;16:1553477. doi: 10.3389/fimmu.2025.1553477 (PMC11931070; doi:10.3389/fimmu.2025.1553477)
Supplement: Supplementary Table 1 — qRT-PCR primers used in this study. [file Table1.docx]

Supplementary Table 1. qRT-PCR primers used in this study

| **Genebank (ID)** | **Genes** | **Forward primer (5’-3’)** | **Reverse primer (5’-3’)** | **Product length (bp)** |
| --- | --- | --- | --- | --- |
| NM_012153.5 | *EHF* | TGATTCTGGAAGGAGGTGGT | ATGTCGAACTCTTGGAAAGGG | 238 |
| [NM_203500](http://www.ncbi.nlm.nih.gov/entrez/query.fcgi?cmd=Search&db=Nucleotide&term=NM_203500) | *Keap1* | CTGGAGGATCATACCAAGCAGG | GGATACCCTCAATGGACACCAC | 220 |
| [NM_000169](http://www.ncbi.nlm.nih.gov/entrez/query.fcgi?cmd=Search&db=Nucleotide&term=NM_000169) | *GLA* | CTGAGGAACCCAGAACTACATCT | GGTAGGCGTCCTTGCCAAT | 117 |
| NM_014599 | *MAGED2* | ACAAAGGTCAATACAAAGGCTCA | GGGCCGAGTATCCTGATTCTC | 117 |
| [NM_001242484](http://www.ncbi.nlm.nih.gov/entrez/query.fcgi?cmd=Search&db=Nucleotide&term=NM_001242484) | *EIF1AD* | GATTGTCAGGGTACTCAGGACC | GATTGTCAGGGTACTCAGGACC | 100 |
